# Supplementary material for: Epigenetic reshaping through damage: promoting cell fate transition by BrdU and IdU incorporation
Source: Cell Biosci. 2024 Jan 16;14:9. doi: 10.1186/s13578-024-01192-x (PMC10792782; doi:10.1186/s13578-024-01192-x)
Supplement: Supplementary file 1 — Additional file 1: Figure S1. A. Schematic diagram of the induction of stage1. B. Morphological changes at barriers1 under indicated conditions. Scale bar 100 $\mu$μm. C. Images of GFP+ colonies taken by fluorescence microscope in situ. Scale bar, 5 mm. D. Morphological changes at barriers1 treated with BrdU, EdU or 5Aza. E. Number of Oct4-GFP + CiPSC colonies generated under BrdU,EdU or 5Aza. n = 3, ∗ ∗ ∗ P < 0.001. F. Morphological changes at distinct time points during induction of CiPSCs treated with BrdU, I + B(IdU + BrdU) or B + E(BrdU + EdU). Scale bar 100 $\mu$μm. G. Number of Oct4-GFP + CiPSC colonies generated under BrdU, I + B or B + E. n = 3, ∗ P < 0.05, ∗ ∗ P < 0.01. Figure S2. A. Representative fibroblast genes, XEN genes and pluripotency genes peaks from RNA-seq for CIP at D8, 14, 20 and 26 with or without BrdU or IdU. B. PCA analysis for RNA-seq data from Cip under indicated conditions. C. Heatmap of XEN genes related RNA-seq for CIP at D8, 14, 20 and 26 with or without BrdU or IdU. D. The R2 correlation coefficient matrix of all versus all RNA-seq datasets as indicated. Figure S3. A. Venn diagrams for overlapping genes in the downregulated groups between BrdU, IdU, and BrdU + IdU versus the control. B. Enriched GO functions in upregulated groups. C. Representative DNA repair genes from RNA-seq for CIP. D.qPCR analysis of the representative DNA repair genes. Figure S4. A. M-bias plot analysis. B. Genomic panorama DNA methylation. Outermost circle: Chromosome karyotype. Se [file 13578_2024_1192_MOESM1_ESM.docx]

**Additional Figure**


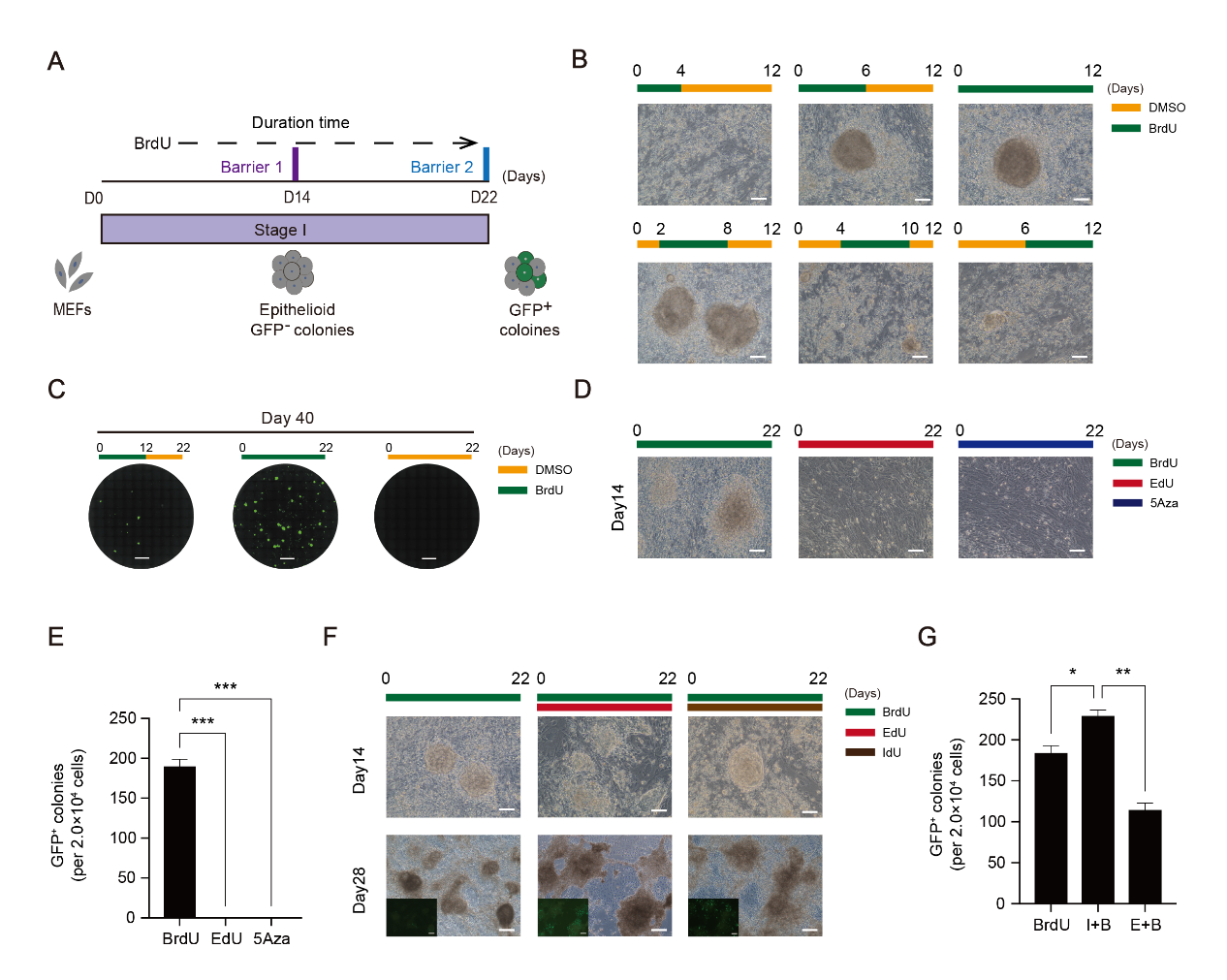


Figure S1

1. Schematic diagram of the induction of stage1
2. Morphological changes at barriers1 under indicated conditions. Scale bar 100$\mu$m
3. Images of GFP+ colonies taken by fluorescence microscope in situ. Scale bar,5mm.
4. Morphological changes at barriers1 treated with BrdU, EdU or 5Aza.
5. Number of *Oct4*-GFP+CiPSC colonies generated under BrdU,EdU or 5Aza. n=3, ∗∗∗P < 0.001.
6. Morphological changes at distinct time points during induction of CiPSCs treated with BrdU ,I+B(IdU+BrdU) or B+E(BrdU+EdU). Scale bar 100$\mu$m
7. Number of *Oct4*-GFP+CiPSC colonies generated under BrdU,I+B or B+E. n=3, ∗P < 0.05, ∗∗P < 0.01.


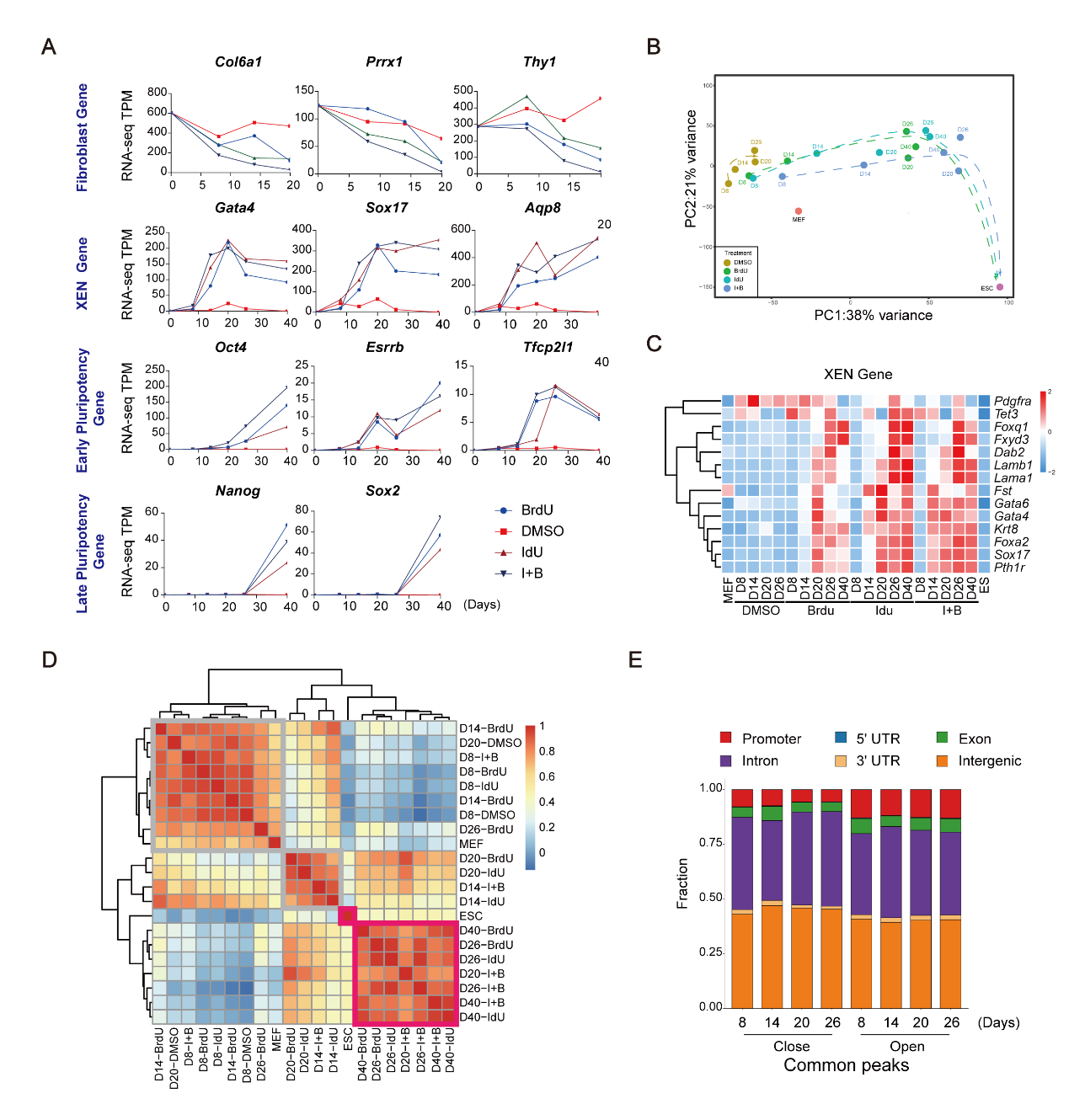


Figure S2

1. Representative fibroblast genes, XEN genes and pluripotency genes peaks from RNA-seq for CIP at D8,14,20 and 26 with or without BrdU or IdU.
2. PCA analysis for RNA-seq data from Cip under indicated conditions
3. Heatmap of XEN genes related RNA-seq for CIP at D8,14,20 and 26 with or without BrdU or IdU.
4. The R2 correlation coefficient matrix of all versus all RNA-seq datasets as indicated


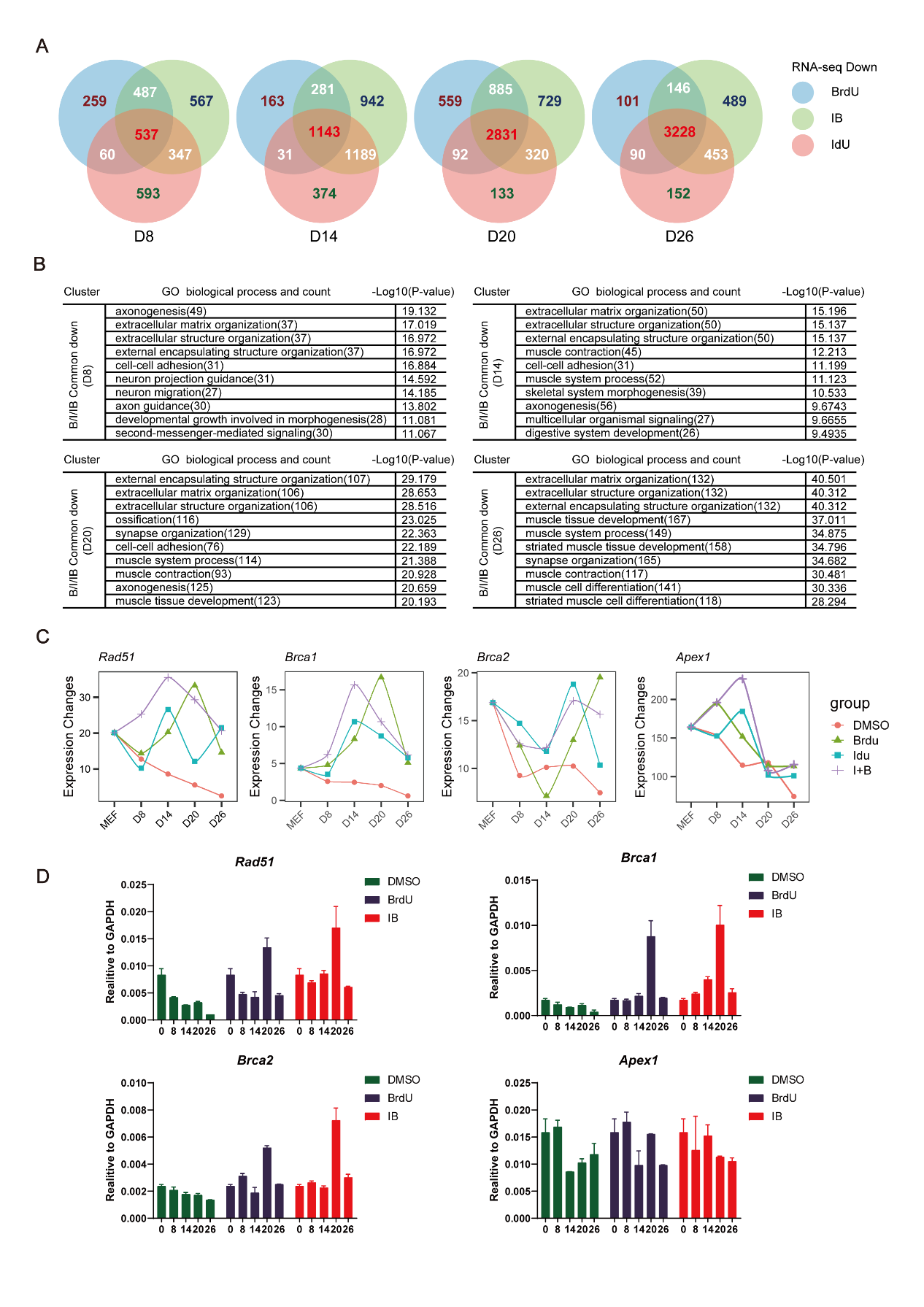


Figure S3

A. Venn diagrams for overlapping genes in the downregulated groups between BrdU, IdU, and BrdU+IdU versus the control.

B. Enriched GO functions in upregulated groups.

C. Representative DNA repair genes from RNA-seq for CIP.

D.qPCR analysis of the representative DNA repair genes.


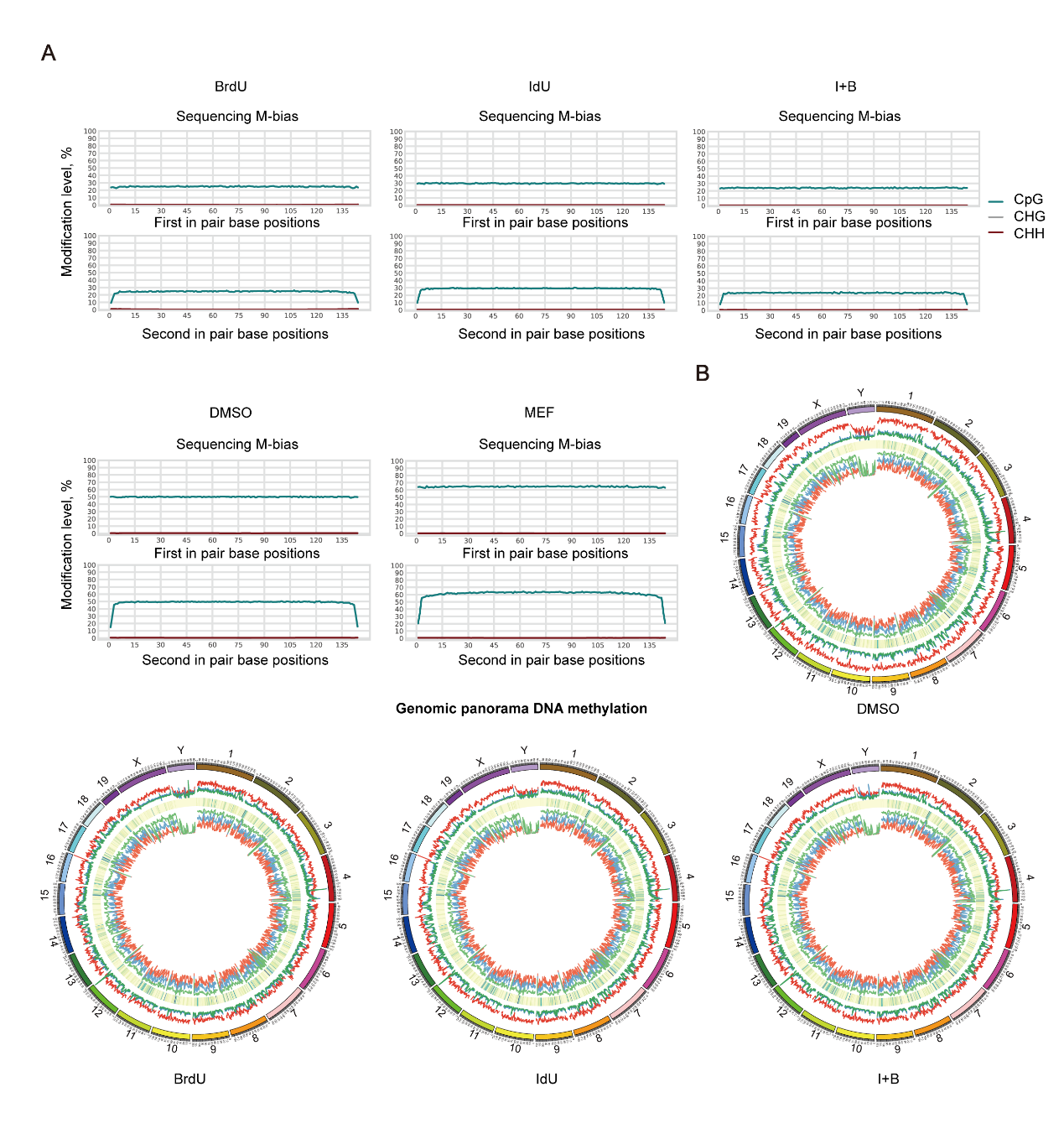


Figure S4

A. M-bias plot analysis

B. Genomic panorama DNA methylation.Outermost circle: Chromosome karyotype.

Second circle: Methylation levels at various genomic positions. The horizontal axis represents genome positions, and the vertical axis represents methylation levels (ranging from 0 to 1, inward to outward direction). Red indicates CpG methylation, blue represents CHG methylation, and green signifies CHH methylation.

Third circle: Gene density, where darker shades of blue indicate higher density.

Innermost circle: Number of methylation sites at different genomic positions. The horizontal axis represents genome positions, and the vertical axis represents the count of methylation sites (increasing from inward to outward). Red indicates CpG sites, blue represents CHG sites, and green represents CHH sites.


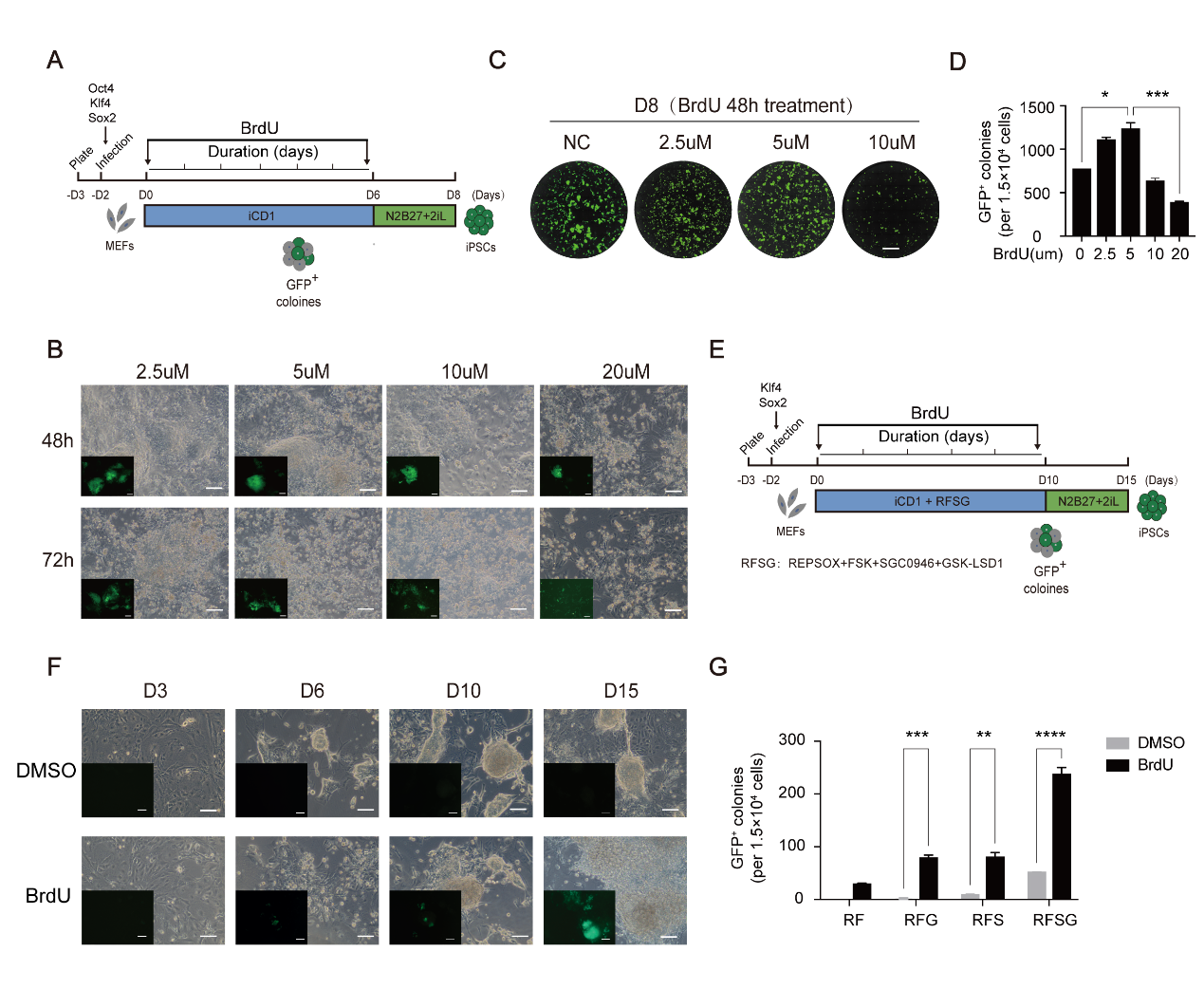


Figure S5

A. Schematic diagram of the induction of iPS from MEFs with OKS

B. Images of GFP+ colonies taken by fluorescence microscope in situ. Scale bar,5mm.

C. Number of *Oct4*-GFP+CiPSC colonies generated under indicated conditions.

D. Morphological changes at OKS treated with different concentrations BrdU. n=3，∗P < 0.05, ∗∗∗P < 0.001.

E. Schematic diagram of the induction of iPS from MEFs with KS

F. Morphological changes at KS treated with or without BrdU

G. Number of *Oct4*-GFP+CiPSC colonies generated under indicated conditions. R,Repsox,F,FSK,S,SGC0946,G,GSK-LSD1.n=3, ∗∗P < 0.01, ∗∗∗P < 0.001 and ∗∗∗∗P < 0.0001.
