# Supplementary material for: Epigenetic reshaping through damage: promoting cell fate transition by BrdU and IdU incorporation
Source: Cell Biosci. 2024 Jan 16;14:9. doi: 10.1186/s13578-024-01192-x (PMC10792782; doi:10.1186/s13578-024-01192-x)
Supplement: Supplementary file 2 — Additional file 2: Table S1. Key resources table. Including: antibodies, chemicals, critical commercial assays, deposited data, experimental models, software and algorithms, primer. [file 13578_2024_1192_MOESM2_ESM.docx]

| REAGENT or RESOURCE | SOURCE | IDENTIFIER |
| --- | --- | --- |
| Antibodies |  |  |
| mouse anti-BrdU | Sigma-Aldrich | B2531 |
| rabbit anti-γ-H2AX | Abcam | ab81299 |
| mouse anti-gata4 | Santa Cruz | sc-25310 |
| rabbit anti-H3K9ac | Abcam | ab4441 |
| rabbit anti-H3K27ac | Abcam | ab4729 |

KEY RESOURCES TABLE

| Chemicals, Peptides, and Recombinant Proteins | | |
| --- | --- | --- |
| Vitamin C | Sigma-Aldrich | 49752; CAS: 66170-10-3 |
| bFGF | PeproTech | GenPept: P09038 |
| CHIR99021 | Synthesized in GIBH | N/A |
| BrdU | Sigma-Aldrich | CAS:59-14-3 |
| RepSox | ChemBest | CAS: 446859-33-2 |
| FSK | ChemBest | CAS:66575-29-9 |
| VPA | Sigma-Aldrich | P4543; CAS: 1069-66-5 |
| AM580 | Tocris | 0760; CAS: 102121-60-8 |
| EPZ5676 | Selleck Chemicals | S7062 |
| DZNeP | Selleck Chemicals | S7120 |
| SGC0946 | Selleck Chemicals | S7079 |
| BMP4 | R&D | 314-BP;GenPept: Q53XC5 |
| Capmatinib | TargetMol | T1963 |
| SGI-1027 | Selleck | S7276；CAS: 1020149-73-8 |
| CM272 | Selleck | S8812； CAS: 1846570-31-7 |
| EdU | Selleck | [S1661](https://www.selleck.cn/products/5-ethynyl-2--deoxyuridine.html)；CAS: 61135-33-9 |
| LIF | Millipore | Cat#ESGE107 |
| GSK-LSD1 | TargetMol | CAS：T22822 |
| KU-55933 | TargetMol | [T2685](https://www.tsbiochem.com/compound/ku-55933)；CAS：587871-26-9 |

Critical Commercial Assays

| ChamQ SYBR qPCR Master Mix | vazyme | Q311-02 |
| --- | --- | --- |
| TruePrep DNA Library Prep Kit V2 for Illumina | vazyme | TD501 |
| Hyperactive universal cut&tag assay Kit for Illumina | vazyme | TD903 |
| TruePrep Index Kit V3 for Illumina | vazyme | TD203 |
| Oxiselect Comet Assay Kit | Cell-Biolabs | STA-351 |
| EpiTect Bisulfite Kit | QIAGEN | 59104 |
| HiPure Gel Pure DNA Mini Kit | Magen Biotech | D2111-03 |
| Deposited Data |  |  |
| RNA-seq data | This paper | GSA: PRJCA019276 |
| ATAC-seq data | This paper | GSA: PRJCA019276 |
| Cut&tag-seq data | This paper | GSA: PRJCA019276 |
| GM seq-data | This paper | GSA: PRJCA019276 |

| Experimental Models: Cell Lines | | |
| --- | --- | --- |
| OG2-ESCs cell line | This paper | N/A |
| CiPSCs cell line | This paper | N/A |
| Platinum-E (Plat-E) | A gift from The Fourth Military Medical University | N/A |

| Experimental Models: Organisms/Strains | | |
| --- | --- | --- |
| OG2 transgenic mice: CBA/CaJ xC57BL/6J | The Jackson Laboratory | Mouse strain datasheet: 004654 |
| 129Sv/Jae mice | Beijing Vital River Laboratory | Mouse strain datasheet: 217 |
| ICR mice | Beijing Vital River Laboratory | Mouse strain datasheet: 201 |

| Software and Algorithms | | |
| --- | --- | --- |
| FlowJo | Ashland | https://www.flowjo.com/solutions/flowjo/ downloads |
| GraphPad Prism 5 | GraphPad Software | https://www.graphpad.com/support/faqid/ 1952/ |
| ZEN 2009 | Zeiss | https://www.zeiss.com.cn/microscopy/products/microscope-software/zen.html#inpagetabs-0 |
| Accuri C6 Plus | BD biosciences | http://www.bdbiosciences.com/us/instruments/ research/cell-analyzers/bd-accuri/m/1294932/ overview |
| Illustrator | Adobe System Software Ireland | https://www.adobe.com/cn/products/illustrator.html |
| image J | National Institutes of Health | https://imagej.nih.gov/ij/download.html |
| cometscore |  | http://rexhoover.com/index.php?id=cometscore |

| Primer (Bisulfite sequencing PCR) | |
| --- | --- |
| Pth1r-iF  Pth1r-iR  Pth1r-oF  Pth1r-oR  Aqp8-iF  Aqp8-iR  Aqp8-oF  Aqp8-oR | GTGGTATTGGGAATTGAATTAG  CCCAAAAATTTACCTTCCAAACT  AGGAAGTTGTGAGTTATTTGA  CAATATTCTAAAATCTTAAACTAAACCC  AGTAGTTGGAGATTTTTAGGGG  TAATAAAACTAATAAACTTTAAAAACCTAA  GAGTTGGGTTATTTGAGTGG  AACAATAAAAACAATATAACACTATT |

|  | |
| --- | --- |
| Primer (qPCR) |  |
| Rad51-QF | AAGTTTTGGTCCACAGCCTATTT |
| Rad51-QR | CGGTGCATAAGCAACAGCC |
| Brca1-QF | CGAATCTGAGTCCCCTAAAGAGC |
| Brca1-QR | AAGCAACTTGACCTTGGGGTA |
| Brca2-QF | ATGCCCGTTGAATACAAAAGGA |
| Brca2-QR | ACCGTGGGGCTTATACTCAGA |
| Apex1-QF | ACGGGGAAGAACCCAAGTC |
| Apex1-QR | GGTGAGGTTTTCTGATCTGGAG |
